# SARS-CoV-2 infection during pregnancy and the risk of adverse maternal outcomes in the Republic of Georgia: a national birth registry-based cohort study

**DOI:** 10.1186/s12884-024-06329-x

**Published:** 2024-02-22

**Authors:** Natia Skhvitaridze, Amiran Gamkrelidze, Tinatin Manjavidze, Tormod Brenn, Charlotta Rylander

**Affiliations:** 1 Department of Community Medicine, UiT The Arctic University of Norway, N-9037 Langnes, Tromsø, PO Box 6050, Norway; 2 National Center for Disease Control and Public Health, 99 Kakheti highway, Tbilisi, Georgia; 3 The University of Georgia, 77a Kostava Street, Tbilisi, Georgia

**Keywords:** Registry-based cohort study, SARS-CoV-2 infection, Adverse maternal health outcomes, Caesarean section, Intensive care unit, Maternal death

## Abstract

**Background:**

Georgia experienced an increase in maternal deaths (MD) during the severe acute respiratory syndrome coronavirus 2 (SARS-CoV-2) pandemic, which warrants further investigation. This study aimed to assess associations between timing of SARS-CoV-2 infection during pregnancy and MD, post-delivery intensive care unit (ICU) admission, and caesarean section (CS) delivery.

**Methods:**

We performed a national birth registry-based cohort study of pregnant women who had completed 22 weeks of gestation and delivered between February 28, 2020, and August 31, 2022. The data were linked to coronavirus disease 2019 (COVID-19) testing, vital, and immunization registries. Pregnant women were classified into three groups: confirmed SARS-CoV-2 infection from conception through 31 days before delivery; confirmed infection within 30 days before or at delivery; and women negative for SARS-CoV-2 infection or without any test results (reference group). Multivariable logistic regression was used to calculate the adjusted odds ratios (aORs) and 95% confidence intervals (CIs).

**Results:**

Among 111,493 pregnant women, 16,751 had confirmed infection during pregnancy, and 7,332 were fully vaccinated against COVID-19 before delivery. Compared to the reference group, those with confirmed infection within 30 days before or at delivery experienced increased odds of MD (aOR: 43.11, 95% CI, 21.99–84.55), post-delivery ICU admission (aOR: 5.20, 95% CI, 4.05–6.67), and CS delivery (aOR: 1.11, 95% CI, 1.03–1.20).

**Conclusions:**

Pregnant women in Georgia with confirmed SARS-CoV-2 infection within 30 days before or at delivery experienced a considerably higher risk of MD and post-delivery ICU admission and a slightly higher risk for CS delivery. Additionally, the results highlighted that most pregnant women were not vaccinated against COVID-19. These findings should alert stakeholders that adherence to public health preventive measures needs to be improved.

**Supplementary Information:**

The online version contains supplementary material available at 10.1186/s12884-024-06329-x.

## Background

At the onset of the coronavirus disease 2019 (COVID-19) pandemic caused by severe acute respiratory syndrome coronavirus 2 (SARS-CoV-2), uncertainty existed regarding potential risks to pregnant women [1, 2]. Comprehensive evidence suggests pregnant women are a vulnerable group with an increased risk of adverse health outcomes following SARS-CoV-2 infection [3–5]. However, significant differences among nations in healthcare sector performance, testing policies, reporting accuracy, and adherence to preventive measures, including vaccination, impacted the official numbers of SARS-CoV-2 infected cases and complicate cross-country comparisons of the impact on population health. Moreover, studies on the adverse effects of SARS-CoV-2 infection in pregnant women have shown varying results owing to differences in study design, selection criteria for study groups, definitions of infections, and detection methods. The most recent evidence indicates that infection during pregnancy increases the risk of pregnancy complications (preterm delivery, caesarean section [CS] delivery, and intensive care unit [ICU] admission) and adverse outcomes (mortality and stillbirth) for both mothers and newborns [4, 6–8]. However, large representative studies on the health effects among pregnant women with and without SARS-CoV-2 infection are limited, and few have investigated the importance of the timing of SARS-CoV-2 infection. Studies that stratified mothers by the timing of infection did not present results on maternal outcomes in the different groups or lack a control group of non-infected women [9].

The first confirmed case of COVID-19 in the Republic of Georgia, a middle-income country, was detected on February 26, 2020. Despite a rapid national pandemic response, COVID-19 cases increased exponentially, and in December 2020, Georgia recorded the highest incidence in Europe [10, 11]. Georgia faced several waves of infection in 2021, the largest of which occurred between January and March 2022. Antenatal care (ANC) and delivery services were considered as essential health services that were to be provided without interruption during the pandemic. Improving maternal and newborn health has been a priority for Georgian public health authorities, who proposed a long-term strategy in 2017 to reduce the maternal mortality (MM) ratio to 12 per 100,000 live births by 2030 [12]. However, MM surged during the pandemic, with MM ratios increasing from 28.9 per 100,000 live births in 2019 to 71.8 per 100,000 live births in 2021 [13]. Georgia implemented mandatory SARS-CoV-2 testing during ANC and delivery. Although the country has several high-quality national health registries, no study has utilized these resources to assess whether pregnant women with SARS-CoV-2 infection experience an increased risk of adverse maternal outcomes compared to non-infected pregnant women. We hypothesized that women with SARS-CoV-2 infection close to or at delivery have a higher risk of adverse maternal outcomes than non-infected women and women with SARS-CoV-2 infection earlier in pregnancy. Therefore, this study aimed to assess the risk of maternal death (MD), post-delivery ICU admission, and CS delivery in relation to SARS-CoV-2 infection during pregnancy among women who had attained a gestational age (GA) of ≥ 22 weeks at the time of delivery.

## Materials and methods

### Data sources

The Georgian Birth Registry (GBR), a nationwide medical birth registry established in 2016 [14], registers all information during ANC, delivery, and subsequent hospital stay for mothers and newborns, and it covered 99.8% of all pregnancies in Georgia in 2021 [13–15]. The LabCov registry, launched in April 2020, electronically records the results of SARS-CoV-2 diagnostic tests, encompassing serological tests (antigen) and molecular tests (reverse transcription polymerase chain reaction), conducted by both private and state laboratories throughout the country. Individuals are registered with LabCov during sample collection and test results must be entered within 24 h of their availability [16, 17]. From June 2020, all pregnant women admitted to birth centers or hospitals were routinely tested for SARS-CoV-2 [17]. Pregnant women in Georgia have eight free-of-charge ANC visits in GA weeks < 13, 18, 26, 30, 34, 36, and 38 [18]. From October 2021, routine testing of pregnant women during ANC visits was implemented. The Immunization Electronic Module, implemented in 2019, registers all vaccinations performed in the country. The Vital Registration System records all deaths in the country and is considered highly complete with 100% coverage. The details of these national digital registration systems administered by the healthcare sector are described elsewhere [14, 15, 19].

### Study population

Given our hypothesis that SARS-CoV-2 infection close to or at the time of delivery increases the risk of adverse maternal outcomes, we included data from all women who had attained GA week ≥ 22 at the time of delivery, as recorded in the GBR. This inclusion criterion was based on the international classification of abortion and delivery [20]. Our study included all women registered in the GBR who delivered between February 28, 2020, and August 31, 2022, totaling 111,493 individuals.

### Exposure and covariates

Information regarding the testing and confirmation of SARS-CoV-2 infection status during pregnancy was obtained from LabCov. Pregnant women were then classified into three exposure groups: no confirmed infection during pregnancy (reference group), which included SARS-CoV-2-negative women and those with no recorded test results; confirmed infection from conception to 31 days before delivery; and confirmed infection within 30 days before or at delivery. The threshold of 30 days was chosen based on previous studies indicating that the virus takes an average of 30 days to clear from the body after a positive test result [21, 22]. Only the positive test result was considered in women with multiple confirmed SARS-CoV-2 infections during pregnancy.

We extracted information about relevant covariates, including sociodemographic characteristics (maternal age, education, and residency), BMI at the first ANC visit, obstetric history (plurality, adherence to ANC, and gestational diabetes), and GA at delivery, from the GBR. Education was classified as primary, secondary, higher, or unknown and residence was categorized as rural or urban. BMI at the first ANC visit was divided into four groups (18.5 kg/m^2^, 18.5–24.9 kg/m^2^, 25–30 kg/m^2^, and > 30 kg/m^2^). Most obstetric history covariates were dichotomized (parity as primiparous or multiparous, plurality as singleton or multiple, and adherence to ANC and gestational diabetes as yes or no), but GA at delivery was divided into four groups (preterm ≤ 36 weeks; early term 37–38 weeks; full term 39–40 weeks; late term ≥ 41 weeks). Being fully vaccinated against COVID-19 was defined as receiving two full doses of vaccine either before SARS-CoV-2 infection (for women with confirmed infection) or at any time during pregnancy (for those with no confirmed infection). SARS-CoV-2 testing prior to pregnancy was limited to high-risk groups, specific professions, contacts, or those with a strong suspicion of infection, leading to inconsistent data on pre-pregnancy infection status. Consequently, this variable was omitted from the analysis owing to its unsystematic collection.

### Outcomes

There were three main maternal outcomes in this study: MD, post-delivery ICU admission, and CS delivery. The standard definition of MD is a death at any time during pregnancy and up to 42 days after pregnancy termination [20]. In this study, we restricted the definition to the death of a pregnant woman who had attained a GA of 22 weeks and up to 42 days after delivery, since delivery was a prerequisite for our study hypothesis. Information on MD was extracted from the Vital Registration System, and information on maternal post-delivery ICU admission and CS delivery was extracted from the GBR. Post-delivery ICU admission was defined as any admission to the ICU (within or outside the location of the birthing facility) after delivery, and CS deliveries included both elective and emergency CS deliveries.

### Statistical analyses

All statistical analyses were performed using STATA, version 17 (StataCorp, TX, USA). Nominal data are presented as frequencies and percentages. Continuous variables are presented as means and standard deviations.

Logistic regression analyses were performed to assess the association of SARS-CoV-2 infection during pregnancy with MD, post-delivery ICU admission, and CS delivery. Directed acyclic graphs (DAGs) were used to identify possible confounding factors in the assumed causal pathways between SARS-CoV-2 infection during pregnancy and MD, post-delivery ICU admission, and CS delivery. The relationships depicted between the variables included in the DAGs were based on previous literature and underlying theories (details in Supplementary material). Based on the DAGs, MD models were adjusted for age, education, BMI at the first ANC visit, parity, gestational diabetes, and COVID-19 vaccination status; post-delivery ICU admission models were adjusted for age, BMI at the first ANC visit, gestational diabetes, and COVID-19 vaccination status; and CS delivery models were adjusted for age, education, BMI at the first ANC visit, parity, and gestational diabetes (Supplementary Figs. 1–3). We also performed a sensitivity analysis for post-delivery ICU admission by excluding MDs and women who underwent CS delivery. Moreover, to ensure that the inclusion of women with no recorded SARS-CoV-2 test results did not bias the results, we performed additional sensitivity analyses in which we restricted the models for all three outcomes to women with recorded SARS-CoV-2 test results in LabCov during pregnancy (Supplementary Table 1). All results are presented as crude and adjusted odds ratios (aORs) with 95% confidence intervals (CIs).

## Results

Among 111,493 pregnant women who gave birth in Georgia from February 28, 2020, to August 31, 2022, 13,800 (12.4%) had confirmed SARS-CoV-2 infection in early pregnancy (from conception through 31 days before delivery), and 2,915 (2.6%) had confirmed infection within 30 days before or at delivery.

Majority of the investigated characteristics showed similarities across the three exposure groups; the mean maternal age was approximately 29 years for all groups, most had secondary education, attended ANC, delivered at full term, and were not fully vaccinated against COVID-19 (Table 1). However, both groups of women with confirmed SARS-CoV-2 infection tended to have certain characteristics more frequently than those in the reference group. These characteristics included a higher likelihood of attaining a higher education level, living in urban areas, attending ANC, being primiparous, and being fully vaccinated against COVID-19. Gestational diabetes was rare and was equally distributed across the exposure groups.

**Table 1:** Characteristics of pregnant women by SARS-CoV-2 infection status. The Georgian Birth Registry, February 28, 2020- August 30, 2022

	No confirmed SARS-CoV-2 infection (reference group) *n* = 94,778*	Confirmed SARS-CoV-2 infection in early pregnancy* n = 13,800*	Confirmed SARS-CoV-2 infection within 30 days before or at delivery *n* = 2,915*
Maternal age, mean (SD)	28.9 (5.90)	29.6 (5.69)	29.4 (5.83)
Education, n (%)			
Primary Secondary Higher Unknown	7,395 (7.8) 38,869 (41.0) 29,922 (31.6) 18,591 (19.6)	441 (3.2) 5,227 (37.9) 5,221 (37.8) 2,911 (21.1)	163 (5.6) 1,161 (39.8) 995 (34.2) 594 (20.4)
Residency, n (%)			
Urban Rural Unknown	68,952 (72.8) 25,750 (27.1) 76 (0.1)	11,047 (80.0) 2,753 (20.0) 0 (0)	2,181 (74.9) 734 (25.1) 0 (0)
BMI at first ANC visit, n (%)			
&lt;18.5 18.5–24.9 25–30 &gt;30	5,965 (6.6) 52,862 (58.5) 20,577 (22.8) 10,926 (12.1)	846 (6.3) 7,924 (58.8) 2,997 (22.3) 1,698 (12.6)	185 (6.6) 1,581 (56.3) 682 (24.3) 361 (12.8)
Parity, n (%)			
Primiparous Multiparous	36,508 (38.5) 58,270 (61.5)	5,621 (40.7) 8,179 (59.3)	1,166 (40.0) 1,749 (60.0)
Plurality, n (%)			
Singleton Multiple	93,167 (98.3) 1,611 (1.7)	13,585 (98.4) 215 (1.6)	2,865 (98.3) 50 (1.7)
Adherence to ANC**, n (%)			
No care Attended	4,400 (4.6) 90,378 (95.4)	313 (2.3) 13,487 (97.7)	85 (2.9) 2,830 (97.1)
Gestational age during first ANC visit, mean (SD)	10.1 (4.64)	9.8 (3.89)	9.6 (3.65)
Gestational diabetes, n (%)			
Yes No	143 (0.2) 94,634 (99.8)	31 (0.2) 13,769 (99.8)	7 (0.2) 2,906 (99.8)
Fully vaccinated***, n (%)			
Yes No	5,084 (5.4) 89,694 (94.6)	1,739 (12.6) 12,057 (87.4)	310 (10.6) 2,605 (89.4)

* Not all numbers add up to the total because of missing observations in the Georgian Birth Registry

** Antenatal care

*** Receiving two full doses of vaccine either before SARS-CoV-2 infection (for women with confirmed infection) or any time during pregnancy (for those with no confirmed infection)

In total, 39 women died during the study period. Of these, 23 (59%) had confirmed SARS-CoV-2 infection during pregnancy, and none were fully vaccinated. Among the 649 women admitted to the post-delivery ICU, 37 (5.7%) were fully vaccinated. Women with SARS-CoV-2 infection within 30 days before or at delivery were more likely to experience MD (0.75% vs. 0.02%) and post-delivery ICU admission (2.57% vs. 0.54%) than those in the reference group (Table 2). Furthermore, compared to the reference group, both groups of women with confirmed infection had a higher likelihood of CS delivery (44.8% and 45.9% vs. 41.9%, Table 2).

**Table 2:** Unadjusted and aORs and 95% CIs for the association between SARS-CoV-2 infection during pregnancy and maternal death, post-delivery intensive care unit admission, and caesarean section delivery

	No confirmed SARS-CoV-2 infection (reference group) *n* = 94,778		Confirmed SARS-CoV-2 infection in early pregnancy* n = 13,800			Confirmed SARS-CoV-2 infection within 30 days before or at delivery *n* = 2,915		
Outcome	n	OR/aOR	n	OR (95%CI)	aOR (95%CI)	n	OR (95%CI)	aOR (95%CI)
Maternal death^a^	16	1.0/1.0	1	0.43 (0.06–3.24)	0.47 (0.06–3.59)	22	45.03 (23.62–85.84)	43.11 (21.99–84.55)
Post-delivery intensive care unit admission^b^	509	1.0/1.0	65	0.88 (0.68–1.14	0.93 (0.71–1.21)	75	4.89 (3.83–6.25)	5.20 (4.05–6.67)
Caesarean section delivery^c^	39,720	1.0/1.0	6,182	1.12 (1.09–1.17)	1.07 (1.03–1.11)	1,337	1.17 (1.09–1.26)	1.11 (1.03–1.20)

aOR – adjusted odds ratio; 95% CI – 95% confidence interval; SARS-CoV-2 – severe acute respiratory syndrome coronavirus 2.

^a^The aOR was adjusted for age, education, parity, body mass index at first antenatal care visit, gestational diabetes, and COVID-19 vaccination.

^b^The aOR was adjusted for age, body mass index at first antenatal care visit, gestational diabetes, and COVID-19 vaccination.

^c^The aOR was adjusted for age, education, parity, body mass index at first antenatal care visit, gestational diabetes, and COVID-19 vaccination.

^*^From conception until 31 days before delivery.

After adjusting for confounding factors, the odds of MD were almost 43 times higher among women with confirmed SARS-CoV-2 infection within 30 days before or at delivery compared to the reference group (aOR, 43.11; 95% CI, 21.99–84.55). In contrast, women with confirmed infection in early pregnancy did not experience higher odds of MD than the reference group (aOR, 0.47; 95% CI, 0.06–3.59). The odds of post-delivery ICU admission were five times higher among women with confirmed SARS-CoV-2 infection within 30 days before or at delivery (aOR, 5.20; 95% CI, 4.05–6.67) compared to the reference group, whereas women with confirmed SARS-CoV-2 infection in early pregnancy had odds of post-delivery ICU admission that were similar to those in the reference group (aOR, 0.93; 95% CI, 0.71–1.21). Notably, the increased odds of post-delivery ICU admission in women with confirmed SARS-CoV-2 infection within 30 days before or at delivery remained high after the exclusion of women with the outcomes of MD (aOR, 4.44; 95% CI, 3.37–5.88) and CS delivery (aOR, 5.39; 95% CI, 3.51–8.27, Supplementary Table 1). Further, women with confirmed SARS-CoV-2 infection within 30 days before or at delivery had 11% higher odds of CS delivery (aOR, 1.11; 95% CI, 1.03–1.20) compared to the reference group (Table 2). Likewise, women with confirmed SARS-CoV-2 infection in early pregnancy experienced 7% higher odds of CS delivery (aOR, 1.07; 95% CI, 1.03–1.11). After restricting the analyses to women with recorded test results in LabCov, the associations between confirmed infection within 30 days before or at delivery and MD (aOR, 36.6; 95% CI, 16.6–80.7), post-delivery ICU admission (aOR, 4.80; 95% CI, 3.68–6.26), and CS delivery (aOR, 1.10; 95% CI, 1.01–1.19) remained strong.

## Discussion

This paper presents results from a national birth registry-based cohort study, conducted in a middle-income country, evaluating the risks of SARS-CoV-2 infection at different times during pregnancy in relation to CS delivery and adverse maternal outcomes. Our results clearly demonstrate that pregnant women with confirmed SARS-CoV-2 infection within 30 days before or at delivery had a substantially increased risk of MD and post-delivery ICU admission. Compared to the reference group, their odds of MD and post-delivery ICU admission were 43 and 5 times higher, respectively. The odds of CS delivery also increased slightly. Notably, women infected in early pregnancy (conception to 31 days before delivery) and attained 22 weeks of gestation before delivery did not exhibit increased odds of MD or post-delivery ICU admission compared to the reference group, suggesting that the timing of SARS-CoV-2 infection during pregnancy is a clear determinant of maternal risk. Few studies have categorized pregnant women based on the timing of SARS-CoV-2 infection, an important factor in our study. Our results have profound implications and should urge stakeholders to accelerate targeted preventive measures in Georgia to avoid infection during pregnancy.

Our results showing increased odds of MD in pregnant women with confirmed SARS-CoV-2 infection within 30 days before or at delivery align with previous studies that reported increased MD during the pandemic compared to the pre-pandemic period [23–25]. However, these studies did not include individual-level data. Our results also align with those of previous original studies, a large-scale multinational study, and systematic reviews of studies with individual-level data on SARS-CoV-2 infection in pregnant women [6, 8, 26–31]. While these studies compared MD risk in infected and uninfected women, we additionally demonstrated that the timing of infection during pregnancy is a clear determinant of risk. Thus, ignoring the timing of infection may obscure the negative effects of SARS-CoV-2 infection. In contrast to our findings, a national registry-based study from Denmark found no association between SARS-CoV-2 infection and MD on studying SARS-CoV-2-positive and -negative pregnant women [32]. Our findings also differ from those of a Nordic study that compared infected pregnant women to pregnant women in the pre-pandemic period [33] and from systematic reviews that covered the early period of the pandemic [26, 34]. However, these results are not surprising, given the disparities in resources between high-income and low- or middle-income countries which can influence the adverse effects of the virus on maternal health. Moreover, some studies included in the systematic reviews were performed in the early phases of the pandemic, when less transmissible variants of the virus were circulating, resulting in fewer infected women. Our results suggest that SARS-CoV-2 infection within 30 days before or at delivery significantly contributed to MD in Georgia during the study period. Reduced quality of care during the first wave of infection when the number of cases increased exponentially might explain negative outcomes. However, other factors like lack of staff due to infection and redeployment of staff to support COVID-19 patients may also explain our results. As healthcare systems and pandemic responses differed across countries, study results from different parts of the world vary.

In our study, the odds of post-delivery ICU admission remained almost 5.5 times higher in women with confirmed SARS-CoV-2 within 30 days before or at delivery compared to the reference group, even after excluding women who experienced MD and CS deliveries. In contrast, SARS-CoV-2 infection during early pregnancy did not increase the odds of post-delivery ICU admission, possibly due to sufficient recovery time before delivery. Contrary to initial reports of no serious negative maternal outcomes [26, 32], studies based on data from the later stages of the pandemic are consistent with our findings of increased odds of post-delivery ICU admission in SARS-CoV-2-positive women around the time of delivery [6–8, 30, 31]. Although most studies agree that SARS-CoV-2 infection during pregnancy increases risk of ICU admission, the strength of this association varies, possibly due to differences in study designs and sample sizes. As with MD, the increased risk of post-delivery ICU admission in SARS-CoV-2-infected pregnant women may be due to medical, organizational, and economic factors. In-depth investigations are needed to untangle the individual contributions of these factors to prevent future delivery complications in these women.

Our analysis also revealed that women with confirmed SARS-CoV-2 infection within 30 days before or at delivery had an 11% higher odds of CS delivery than those in the reference group. Additionally, women with confirmed SARS-CoV-2 infection in early pregnancy had slightly increased odds of CS delivery compared with the reference group. Our results are in line with those of a recent meta-analysis indicating a 16% increased risk of CS delivery in SARS-CoV-2-positive women compared to SARS-CoV-2-negative women [8], although other studies have reported no association between SARS-CoV-2 infection in pregnancy and CS delivery [6, 35]. Thus, the association between SARS-CoV-2 infection and CS delivery may vary across countries owing to different clinical management approaches for COVID-19-infected pregnancies.

Another important aspect revealed in our study was the low proportion of pregnant women in Georgia who were fully vaccinated against COVID-19. The preventive effect of vaccination against the development of severe disease and death is unquestionable [36–38]. Recent studies have indicated the significant benefit of COVID-19 vaccination for pregnant women, similar to the general population [37–40]. Although we did not assess the risk of maternal outcomes according to vaccination status, 59% of the women who died in our study were SARS-CoV-2-positive and none were vaccinated against COVID-19. Moreover, only 5.7% of pregnant women admitted to the ICU were vaccinated. These observations underscore the need for future research on the influence of vaccination status on adverse maternal outcomes in SARS-CoV-2 infected pregnant women in Georgia.

The main strength of this study lies in the utilization of the GBR, a national population-based birth registry. Its use minimized selection bias, because registration in the GBR is mandatory by law. MDs were extracted from the Vital Registration System, which has close to 100% coverage. The national testing strategy of frequently and routinely testing pregnant women for SARS-CoV-2 significantly improved the accuracy of our exposure classification and determination of the timing of infection. However, this study had several limitations. Given our study hypothesis that pregnant women are at a higher risk of adverse maternal outcomes when infected with SARS-CoV-2 close to or at delivery, we only included women who attained 22 weeks of gestation. Hence, women who died before GA week 22 were not eligible for our study because delivery before GA week 22 was defined as abortion. This may have affected our results. Moreover, from the beginning of the pandemic until the implementation of routine testing strategies, a considerable proportion of women with undetected SARS-CoV-2 infection in early pregnancy may have been misclassified as part of the reference group. Even after routine testing was implemented during ANC, pregnant women with COVID-19 that resolved between ANC visits would have remained undetected. As shown in other studies, it was not possible to overcome the potential misclassification of participants who were infected but were never tested [41], which may have affected our results. Furthermore, lack of information on the viral variants, infection status prior to pregnancy, severity of SARS-CoV-2 infection, socioeconomic status, and ethnicity could potentially confound the relationship between SARS-CoV-2 infection and maternal outcomes. Moreover, gestational diabetes is rare and may be under-reported. Finally, our results showed the impact of SARS-CoV-2 infection on adverse maternal health outcomes in pregnant women; however, we had no information regarding the specific causes of death. Further in-depth study is recommended after clinical audits have been finalized and related information on all MDs during the study period has been collected.

## Conclusion

In Georgia, pregnant women with SARS-CoV-2 infection detected within 30 days before or at delivery had a significantly higher risk of MD and post-delivery ICU admission, along with a modest increase in CS delivery compared to uninfected women. Infection earlier in pregnancy did not increase these risks. As most participants were unvaccinated, these results highlight the urgent need for public health efforts to promote COVID-19 preventive measures among pregnant women.

### Electronic supplementary material

Below is the link to the electronic supplementary material.

Supplementary Material 1

## Data Availability

According to Georgian legislation, data from the central health registries can be used for research purposes if personal data are not shared. Appropriate ethical and legal approval are required to meet the criteria for access before requesting data, and only anonymized data can be shared. Researchers can contact the National Center for Disease Control and Public Health in Georgia (ncdc@ncdc.ge) or the corresponding author (natia.skhvitaridze@uit.no) for assistance on how to apply for access to data from central health registries.

## Abbreviations

SARS-CoV-2: severe acute respiratory syndrome coronavirus 2
COVID-19: coronavirus disease 2019
CS: caesarean section
ICU: intensive care unit
ANC: antenatal care
MD: maternal death
MM: maternal mortality
GA: gestational age
GBR: Georgian Birth Registry
BMI: body mass index
DAGs: directed acyclic graphs
aORs: adjusted odds ratios
CIs: confidence intervals
