# Supplementary material for: SARS-CoV-2 infection during pregnancy and the risk of adverse maternal outcomes in the Republic of Georgia: a national birth registry-based cohort study
Source: BMC Pregnancy Childbirth. 2024 Feb 22;24:156. doi: 10.1186/s12884-024-06329-x (PMC10882809; doi:10.1186/s12884-024-06329-x)
Supplement: Supplementary file 1 — Supplementary Material 1 [file 12884_2024_6329_MOESM1_ESM.docx]

**Supplementary material**

**SARS-CoV-2 infection during pregnancy and the risk of adverse maternal outcomes in the Republic of Georgia: a national birth registry-based cohort study**

Submitted to the BMC Pregnancy and Childbirth

Natia Skhvitaridze (ORCID 0000-0002-0312-4151)^1,2,3^, Amiran Gamkrelidze^3^, Tinatin Manjavidze (0000-0003-1340-6256)^1,2,3^, Tormod Brenn (ORCID 0000-0003-3717-8323)^1^, Charlotta Rylander (ORCID 0000-0002-5056-9452)^1^

**Affiliations**

^1^Department of Community Medicine, UiT The Arctic University of Norway, PO Box 6050 Langnes

N-9037 Tromsø, Norway

^2^National Center for Disease Control and Public Health, Tbilisi, 99 Kakheti highway, Georgia

^3^The University of Georgia, Tbilisi, 77a Kostava Street, Georgia

Corresponding author: Natia Skhvitaridze, [natia.skhvitaridze@uit.no](mailto:natia.skhvitaridze@uit.no)

In this paper direct acyclic graphs (DAGs) were used to identify confounding factors for the presumed causal relationship between Severe Acute Respiratory Syndrome Coronavirus 2 (SARS-CoV-2) infection and maternal mortality (MM), maternal post-delivery intensive care unit (ICU) admission, and cesarean section (CS) delivery.

DAGs are useful tool for visualizing assumed relationships between exposures, outcomes and covariates [1]. In the figures below, variables indicated with pink circles are identified confounders and blue circles indicate mediating variables. We included some unobserved variables as well, which are indicated as grey circles.

The assumptions for the causal effect of SARS-CoV-2 infection on MM is described in the DAG presented in supplementary figure 1. COVID-19 infection is a prerequisite for severe COVID-19, which in turn increases the risk of MM [2, 3]. COVID vaccination reduces the risk of severe COVID-19 [4, 5]. Further, we assumed that age, education, residency, BMI at first ANC visit, parity, and gestational diabetes impacted the possibility of being fully vaccinated against COVID-19, as well as adherence to non-pharmaceutical interventions (NPIs) such as use of face masks and social distancing [6-9]. Moreover, gestational diabetes increases the risk of severe COVID-19, as indicated by the arrow from gestational diabetes to severity [10]. Further, severe COVID-19 increases the risk of preeclampsia, post-delivery ICU admission, and CS delivery, and in turn, post-delivery ICU admission, CS, hemorrhage, and preeclampsia are risk factors for MM [11-13]. Based on these assumptions, the minimal sufficient set of variables to include in the models to control confounding and estimate the total effect of SARS-CoV-2 infection on MM includes age, education, parity, BMI at first ANC visit, gestational diabetes, and COVID-19 vaccination status.


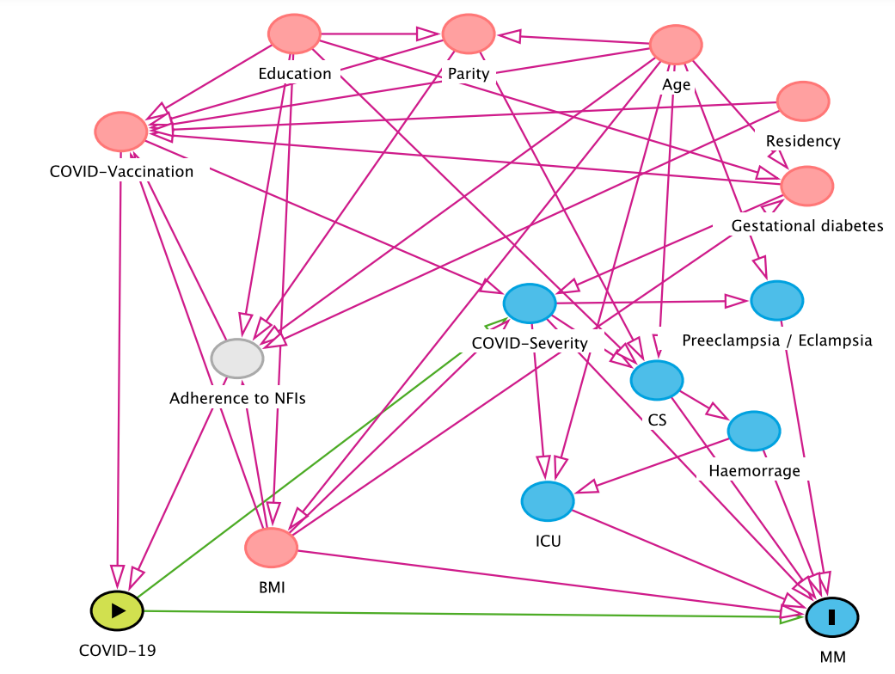
Supplementary Figure 1: Directed acyclic graph indicating covariates and mediators of relationship between SARS-CoV-2 infection and maternal mortality (MM)

The DAG outlined in supplementary figure 2 assumes a causal effect of SARS-CoV-2 infection on post-delivery ICU admission. As was already stated, severe COVID-19 requires SARS-CoV-2 infection, which is also a risk factor for post-delivery ICU admission. COVID-19 vaccination reduces the risk of severe COVID-19 [4]. Further, we assumed that age, education, residency, BMI at first ANC visit, parity, and gestational diabetes impact the possibility of being vaccinated, as well as the adherence to non-pharmaceutical interventions (NPIs) such as use of face masks and social distancing [6-9, 14]. Moreover, gestational diabetes increases the risk of severe COVID-19 as indicated by the arrow from gestational diabetes to severity. SARS-CoV-2 infection also increases the risk of preeclampsia. Based on the existing knowledge and several assumptions, the minimal sufficient set of variables to include in the model to control confounding and estimate the total effect of SARS-CoV-2 infection on post-delivery ICU transfer includes age, BMI at first ANC visit, gestational diabetes, and COVID-19 vaccination status.

*Supplementary Figure 2: Directed acyclic graph indicating covariates and mediators on relationship between SARS-CoV-2 infection and post-delivery intensive care unit (ICU) admission*

Assumptions for the causal effect of SARS-CoV-2 infection on CS delivery is presented in the DAG in supplementary figure 3. After elaborating assumptions, the minimal sufficient set of variables to include in the models to control for confounding and estimate the total effect of SARS-CoV-2 infection on CS includes age, education, parity, BMI at first ANC visit, gestational diabetes and COVID-19 vaccination status [14].

Supplementary Figure 3: Directed acyclic graph indicating covariates and mediators on relationship between SARS-COV-2 infection and caesarean section (CS) delivery

References:

1. Tennant PWG. Use of directed acyclic graphs (DAGs) to identify confounders in applied health research: review and recommendations. International Journal of Epidemiology. 2020;50(2):620-32. doi:10.1093/ije/dyaa213

2. Villar J. Maternal and Neonatal Morbidity and Mortality Among Pregnant Women with and Without COVID-19 Infection. The INTERCOVID Multinational Cohort Study. JAMA Pediatrics. 2021;175(8):817-26. doi:https://doi.org/10.1001/jamapediatrics.2021.1050

3. Metz TD. Association of SARS-CoV-2 Infection with Serious Maternal Morbidity and Mortality From Obstetric Complications. American Medical Association (JAMA). 2022. doi:https://doi.org/10.1001/jama.2022.1190

4. Tenforde MW. Long-term Protection Associated with COVID-19 Vaccination and Prior Infection. JAMA. 2022;328(14):1402-4. doi:10.1001/jama.2022.14660

5. Watanabe A. Peripartum Outcomes Associated With COVID-19 Vaccination During Pregnancy. American Medical Association (JAMA). 2022. doi:https://doi.org/10.1001/jamapediatrics.2022.3456

6. Admon AJ. Consensus elements for observational research on COVID-19-related long-term outcomes. Medicine. 2022;101(46). doi:10.1097/MD.0000000000031248

7. Anderson MR. Body Mass Index and Risk for Intubation or Death in SARS-CoV-2 Infection - A Retrospective Cohort Study. Annals of Internal Medicine. 2020. doi:https://doi.org/10.7326/M20-3214

8. Kompaniyets L. Body Mass Index and Risk for COVID-19–Related Hospitalization, Intensive Care Unit Admission, Invasive Mechanical Ventilation, and Death — United States, March–December 2020. MMWR Morb Mortal Wkly Rep. 2021. doi:http://dx.doi.org/10.15585/mmwr.mm7010e4

9. Sawadogo W. Overweight and obesity as risk factors for COVID-19-associated hospitalisations and death: systematic review and meta-analys. BMJ Nutr Prev Health. 2022. doi:10.1136/bmjnph-2021-000375

10. Radan A. Gestational diabetes is associated with SARS-CoV-2 infection during pregnancy: A case-control study. Diabetes & Metabolism. 2022;48(4). doi:https://doi.org/10.1016/j.diabet.2022.101351

11. Tasew A. Obstetrics mortality and associated factors in intensive care unit of Addis Ababa public hospital in, 2020/21: A hospital based case control study. Ann Med Surg (Lond). 2022. doi:10.1016/j.amsu.2022.104458

12. de Amorim MMR. Risk factors for maternal death in patients with severe preeclampsia and eclampsia. Revista Brasileira de Saúde Materno Infantil 2001. doi: https://doi.org/10.1590/S1519-38292001000300004

13. De Barros J. Factors associated with severe maternal outcomes in patients with eclampsia in an obstetric intensive care unit. A cohort study. Medicine. 2021;100(38). doi:10.1097/MD.000000000002731

14. Saccone G. Maternal and perinatal complications according to maternal age: A systematic review and meta-analysis. Int J Gynaecol Obstet. 2022;159(1):43-55. doi:10.1002/ijgo.14100

Supplementary Table 1. Crude and adjusted odds ratios (ORs) and 95% confidence intervals (CI) for the association between COVID-19 and maternal post-delivery intensive care unit (ICU) admission in women who survived pregnancy and 42 days after delivery and in women who survived and delivered vaginally

.

|  | **No confirmed SARS-CoV-2 infection (reference group)** | | | **Confirmed SARS-CoV-2 infection in early pregnancy*** | | | | **SARS-CoV-2 infection within 30 days before or at delivery** | | |
| --- | --- | --- | --- | --- | --- | --- | --- | --- | --- | --- |
| **Outcome** | **n** | **ICU, n** | **OR (95% CI)** | **n** | **ICU, n** | **OR (95% CI)** | **aOR (95% CI)** | **n** | **ICU, n** | **OR/aOR** |
| Survivors^[[1]](#footnote-1)^ | 94,762 | 503 | 1.0/1.0 | 13,799 | 65 | 0.89 (0.68-1.15) | 1.00 (0.77-1.31) | 2,893 | 59 | 4.44 (3.37-5.88) |
| Vaginal deliveries and survivors^[[2]](#footnote-2)^ | 55,054 | 177 | 1.0/1.0 | 7,617 | 27 | 1.10 (0.74-1.65) | 1.20 (0.79-1.82) | 1,573 | 25 | 5.39 (3.51-8.27) |

1. aOR – adjusted odds ratio; 95% CI – 95% confidence intervals; SARS-CoV-2 – Severe Acute Respiratory Syndrome Coronavirus 2

   The aOR was adjusted for age, education, place of residency, parity, body mass index at first antenatal care visit, gestational diabetes, COVID-vaccination [↑](#footnote-ref-1)
2. The aOR was adjusted for age, education, place of residency, parity, body mass index at first antenatal care visit, gestational diabetes, COVID-vaccination

   *From conception until 31 days before delivery [↑](#footnote-ref-2)
